# NAIP/NLRC4 inflammasome activation in MRP8^+^ cells is sufficient to cause systemic inflammatory disease

**DOI:** 10.1038/s41467-017-02266-w

**Published:** 2017-12-20

**Authors:** Randilea D. Nichols, Jakob von Moltke, Russell E. Vance

**Affiliations:** 1 Division of Immunology and Pathogenesis, University of California, Berkeley, 94720 California USA; 2 Howard Hughes Medical Institute, University of California, Berkeley, 94720 California USA; 3 Cancer Research Laboratory, University of California, Berkeley, 94720 California USA; 4 Immunotherapeutics and Vaccine Research Initiative, University of California, Berkeley, 94720 California USA; 5 Present Address: Department of Immunology, University of Washington, Seattle, 98109 Washington USA

## Abstract

Inflammasomes are cytosolic multiprotein complexes that initiate protective immunity in response to infection, and can also drive auto-inflammatory diseases, but the cell types and signalling pathways that cause these diseases remain poorly understood. Inflammasomes are broadly expressed in haematopoietic and non-haematopoietic cells and can trigger numerous downstream responses including production of IL-1β, IL-18, eicosanoids and pyroptotic cell death. Here we show a mouse model with endogenous NLRC4 inflammasome activation in *Lysozyme2* ^+^ cells (monocytes, macrophages and neutrophils) in vivo exhibits a severe systemic inflammatory disease, reminiscent of human patients that carry mutant auto-active NLRC4 alleles. Interestingly, specific NLRC4 activation in *Mrp8* ^+^ cells (primarily neutrophil lineage) is sufficient to cause severe inflammatory disease. Disease is ameliorated on an *Asc* ^−/−^ background, and can be suppressed by injections of anti-IL-1 receptor antibody. Our results provide insight into the mechanisms by which NLRC4 inflammasome activation mediates auto-inflammatory disease in vivo.

## Abstract (web summary)

Inflammasomes are protein complexes induced by pathogens for the secretion of pro-inflammatory cytokines IL-1β and IL-18 in immune cells. Here the authors show, using a new mouse model, that aberrant NLRC4 and ASC-dependent inflammasome activation in neutrophils contributes to systemic inflammation.

## Introduction

The innate immune system detects infection using pattern recognition receptors, such as Toll-like receptors (TLR) and cytosolic nucleotide-binding domain, leucine-rich repeat containing proteins (NLR)^1, 2^. Upon pathogen recognition, certain NLRs form cytosolic multiprotein complexes called inflammasomes that function to activate a downstream protease, Caspase-1 (CASP1)^3, 4^. CASP1 initiates inflammation, at least in part, by processing interleukin-1β (IL-1β) and IL-18 into their secreted and active forms. Active CASP1 also cleaves and activates a pore-forming protein called Gasdermin D^5, 6^, thereby inducing cytokine release and a lytic cell death termed pyroptosis^7, 8^. It has been proposed that pyroptotic death is a ‘pro-inflammatory’ form of cell death that is associated with the release of inflammatory mediators^9^. However, the intrinsic inflammatory effects of pyroptosis have been difficult to dissociate from the concurrent release of pro-inflammatory cytokines, such as IL-1β and IL-18.

There are several different inflammasomes, each of which is responsive to unique ligands or stimuli^3, 4^. Activation of the NLR family, CARD domain-containing protein 4 (NLRC4) inflammasome requires NLR family, apoptosis inhibitory proteins (NAIP), which bind specific bacterial ligands and co-assemble with NLRC4^10, 11^. Studies have established that NAIP5 specifically detects the C-terminus of flagellin^12^. Importantly, this region of flagellin is not sufficient to activate TLR5, another innate immune flagellin sensor^12^. Though the CARD domain of NLRC4 can interact directly with CASP1, NLRC4 also recruits CASP1 via the adaptor protein, apoptosis-associated speck-like protein containing a CARD (ASC). *Asc* ^−/−^ macrophages have decreased IL-1β and IL-18 processing but still initiate NLRC4-induced pyroptosis^13, 14^.

The NAIP/NLRC4 inflammasomes presumably evolved to initiate protective immune responses during bacterial infection. Indeed, NAIP/NLRC4 deficiency results in variable degrees of susceptibility to a wide range of bacteria^15, 16^. Conversely, inappropriate NLRC4 inflammasome activation can result in pathology, or even death, in both mice and humans^17–23^. However, the mechanisms by which chronic NLRC4 activation causes pathology remain poorly understood. For example, it is not clear whether IL-1β, IL-18 and/or pyroptotic cell death drive NLRC4-induced disease in vivo. In addition, given that NLRC4 is functional in multiple cell types, including haematopoietic and intestinal epithelial cells, it remains unclear through which cell types NLRC4 activation can drive pathology. A major limitation has been the lack of mouse models that recapitulate NLRC4-driven auto-inflammatory disease via cell-type specific activation of endogenous NLRC4. Kitamura et al.^19^ reported that transgenic mice expressing a constitutively active NLRC4 variant (H443P) develop an auto-inflammatory disease. However, since these transgenic mice overexpress a mutant NLRC4 allele under the control of a non-native (invariant chain) promoter, it is not clear whether persistent activation of endogenous NLRC4 might also produce disease. In addition, it remains unclear in which cell types NLRC4 activation will drive disease.

Prior in vivo studies have primarily used bacterial infection to activate the endogenous NAIP/NLRC4 inflammasome (reviewed in refs. ^15, 16^). Since bacterial infections typically activate numerous innate immune pathways, including TLRs, it has been difficult to separate the effects of NAIP/NLRC4 activation from the downstream pro-inflammatory effects of TLR activation. Consequently, it is not clear whether activation of endogenous wild-type NLRC4 alone would be sufficient to drive inflammation in vivo. For example, induction of IL-1β is generally thought to require priming signals to induce expression of pro-IL-1β prior to its processing by CASP1 downstream of NAIP/NLRC4^24–26^. Thus, NLRC4 activation in the absence of priming might be insufficient to induce inflammation. As one approach to study the priming-independent effects of NAIP/NLRC4 activation in vivo, we and others have selectively activated NLRC4 using ‘FlaTox’, a recombinant flagellin fusion protein that enters cells and activates NLRC4^11, 18, 27, 28^. Experiments with FlaTox demonstrated that NAIP/NLRC4 activation in vivo can cause pathology in the absence of ‘priming’ or inflammasome-induced IL-1β/-18^18^. However, FlaTox has acute lethal effects that make it difficult to model the chronic effects of inappropriate NLRC4 activation, such as those observed in patients with NLRC4 gain-of-function mutations.

We previously found that expression of the C-terminus of bacterial flagellin from a mammalian promoter is sufficient to activate the endogenous NAIP/NLRC4 inflammasome in macrophages in vitro^12^. Here we report the generation of a genetically engineered mouse that inducibly expresses the C-terminal 166 amino acids of *Legionella pneumophila* flagellin, fused to ovalbumin. An advantage of these mice is that they permit the selective activation of the endogenous NAIP/NLRC4 inflammasome without the concomitant provision of additional exogenous ‘priming’ signals. Mice in which endogenous NLRC4 is specifically and selectively activated in *Lysozyme2* ^+^ cells (monocytes, macrophages, neutrophils and select dendritic cell populations^29, 30^) have a marked inflammatory disease characterised by systemic neutrophilia, weight loss, and hind limb joint swelling. The disease is NLRC4-dependent and is ameliorated on the *Asc* ^−/−^ background, suggesting a dominant function for ASC-dependent cytokines and a minimal function for pyroptosis. Consistent with this interpretation, neutrophil levels and disease symptoms decrease after blockade of the IL-1 receptor. Interestingly, severe disease, including joint swelling, is recapitulated by NLRC4 activation selectively in neutrophils and a small subset of monocytes (using MRP8-Cre^29, 31, 32^), but the same severe disease symptoms are not induced upon selective inflammasome activation in dendritic cells, tissue macrophages and a small subset of monocytes (using CD11c-Cre^29, 33^). Disease that arises after MRP8-Cre-induced NLRC4 activation is also ameliorated by anti-IL-1 receptor blockade. These results suggest that neutrophil-dependent IL-1 is a major driver of inflammasome-dependent auto-inflammatory disease in vivo.

## Results

### Genetic system for cell-specific inflammasome activation

**Fig. 1:**
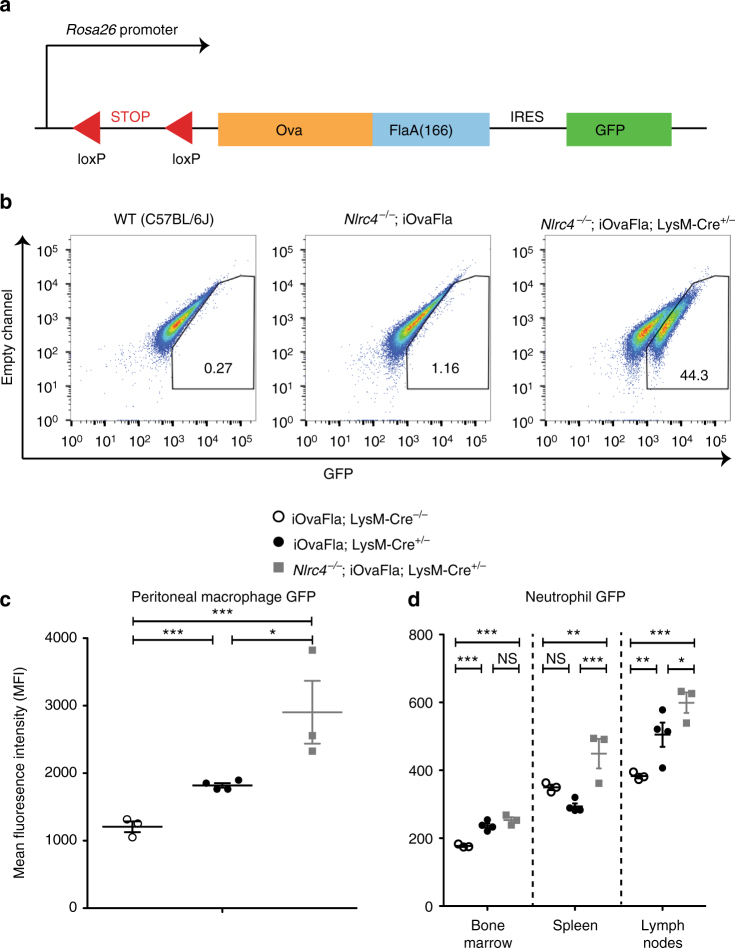
Genetic system for inducible NLRC4 inflammasome activation in vivo. **a** Schematic showing iOvaFla transgene insertion in the *Rosa26* locus. **b** Flow cytometry analysis of bone marrow derived macrophages (BMMs) cultured from WT, *Nlrc4* ^−/−^; iOvaFla and *Nlrc4* ^−/−^; iOvaFla; LysM-Cre^+/−^ mice (representative images from three biological replicates), **c** peritoneal macrophages (CD11b^+^ F4/80^+^) from iOvaFla; LysM-Cre^−/−^, iOvaFla; LysM-Cre^+/−^, and *Nlrc4* ^−/−^; iOvaFla; LysM-Cre^+/−^ mice, and **d** neutrophils (CD11b^+^ Ly6G^Hi^, Ly6C^Lo^) from the bone marrow, spleens and lymph nodes. Data in **b**–**d** are representative of two independent experiments. Error bars are s.d. Results were analysed with a two-way ANOVA and Bonferroni post-tests; *p < 0.05, **p < 0.01, ***p < 0.001

To address the physiological consequences of chronic cell type specific NAIP/NLRC4 activation in vivo, we generated a genetically targeted ‘iOvaFla’ mouse that expresses, under Cre-inducible control, a gene encoding chicken ovalbumin (Ova) (lacking its signal sequence) fused to the C-terminal 166 amino acids of *Legionella pneumophila* flagellin (Fla). Ova was included to permit the eventual tracking of adaptive immune responses in these mice, but this was not part of the present study. The gene encoding the iOvaFla fusion protein was inserted into the ubiquitously expressed *Rosa26* locus downstream of a loxP-flanked transcriptional STOP cassette^34^ to prevent iOvaFla expression until Cre recombinase is expressed (Fig. 1a). An IRES-GFP reporter was also inserted downstream of the iOvaFla gene fusion to allow us to visualise cells expressing iOvaFla. These mice were created on a C57BL/6J background, competent for NAIP/NLRC4 components, unless otherwise indicated in figures.

To test for proper production of the iOvaFla-IRES-GFP construct, we crossed the iOvaFla mice onto a *Nlrc4* ^−/−^ background (to prevent any NLRC4-dependent cell death) and to *Lysozyme2*-Cre (also called LysM-Cre) transgenic mice (to induce iOvaFla-IRES-GFP expression in myeloid cells). Bone marrow-derived macrophages (BMM) were differentiated from wild-type C56BL/6J (B6) mice, *Nlrc4* ^−/−^; iOvaFla mice, and *Nlrc4* ^−/−^; iOvaFla; LysM-Cre^+/−^ mice. As expected, ~44% of *Nlrc4* ^−/−^; iOvaFla^+/−^; LysM-Cre^+/−^ BMMs were GFP^+^, compared to ≤1% of B6 or Cre-negative *Nlrc4* ^−/−^; iOvaFla BMMs (Fig. 1b). As further confirmation of Cre-inducible iOvaFla-IRES-GFP expression, we transduced B6, iOvaFla, and *Nlrc4* ^−/−^; iOvaFla BMMs with either an empty retroviral vector, a GFP expression vector as a transduction efficiency control, or a vector expressing only Cre recombinase (Supplementary Fig. 1a). BMMs transduced with empty vector exhibited minimal GFP induction, whereas BMMs transduced with the control GFP vector all exhibited a significant population (27–33%) of GFP^+^ cells. *Nlrc4* ^−/−^; iOvaFla BMMs transduced with the Cre recombinase vector also exhibited a robust GFP^+^ population (~27%), whereas <5% of Cre-transduced iOvaFla macrophages were GFP^+^ (Supplementary Fig. 1a). The relative lack of GFP expression specifically in NLRC4 inflammasome-competent BMMs is expected due to the loss of GFP expressing cells as a result of NLRC4-induced pyroptotic cell death, as we have previously described^12^.

We also assessed GFP expression in ex vivo isolated resident peritoneal macrophages from iOvaFla mice. Though NLRC4 inflammasome-competent iOvaFla; LysM-Cre^+/−^ macrophages expressed GFP at a higher median fluorescence intensity (MFI) as compared to their Cre-negative littermates, expression levels were lower as compared to *Nlrc4* ^−/−^; iOvaFla; LysM-Cre^+/−^ mice (Fig. 1c, Supplementary Fig. 1c). These results were confirmed by GFP immunoblot (Supplementary Fig. 1b). The difference in GFP levels between NLRC4 inflammasome-competent and *Nlrc4* ^−/−^ macrophages is consistent with the expected loss of GFP^+^ macrophages via pyroptosis after NLRC4 inflammasome activation by iOvaFla.

Unlike macrophages, it has been controversial whether neutrophils undergo pyroptosis upon NLRC4 activation^35, 36^. *Salmonella enterica* serovar Typhimurium infected bone marrow neutrophils and ex vivo peritoneal neutrophils release NLRC4-dependent mature IL-1β but were reported to not undergo pyroptosis as measured by lactate dehydrogenase release^35^. Ryu et al.^36^ reported pyroptosis of lung neutrophils but only in the absence of NADPH oxidase 2. We therefore sought to examine GFP levels in bone marrow, splenic and lymph node neutrophils from our iOvaFla mice as an indirect but in vivo assay for pyroptosis. The auto-fluorescence of neutrophils made definitive conclusions difficult. Nevertheless, in all tissues, *Nlrc4* ^−/−^; iOvaFla; LysM-Cre^+/−^ neutrophils consistently had the highest GFP MFI, whereas Cre-negative littermates had the lowest apparent GFP MFI (green fluorescence in Cre-negative mice is presumably due to background auto-fluorescence) (Fig. 1d, Supplementary Fig. 1d). NLRC4-dependent loss of GFP was only significant (*p* < 0.001 and *p* < 0.05, respectively, by two-way ANOVA and Bonferroni post-test) in neutrophils from spleen and lymph nodes and not in bone marrow neutrophils. One interpretation of these data is that splenic or lymph node neutrophils, but not bone marrow neutrophils, can undergo NLRC4-dependent pyroptosis in vivo. However, because measurement of GFP MFI is a highly indirect assay of pyroptosis, other explanations are also possible. For example, NLRC4 activation may hinder the development or homing of splenic or lymph node neutrophils. Importantly, we did not observe LysM-Cre-induced GFP expression in other cell types (e.g., B cells and T cells) (Supplementary Fig. 1e). Thus, taken together, the above data demonstrate LysM-Cre-dependent induction of the iOvaFla-IRES-GFP construct in macrophages and neutrophils in vivo.

### Myeloid cell NLRC4 activation causes systemic inflammation

**Fig. 2:**
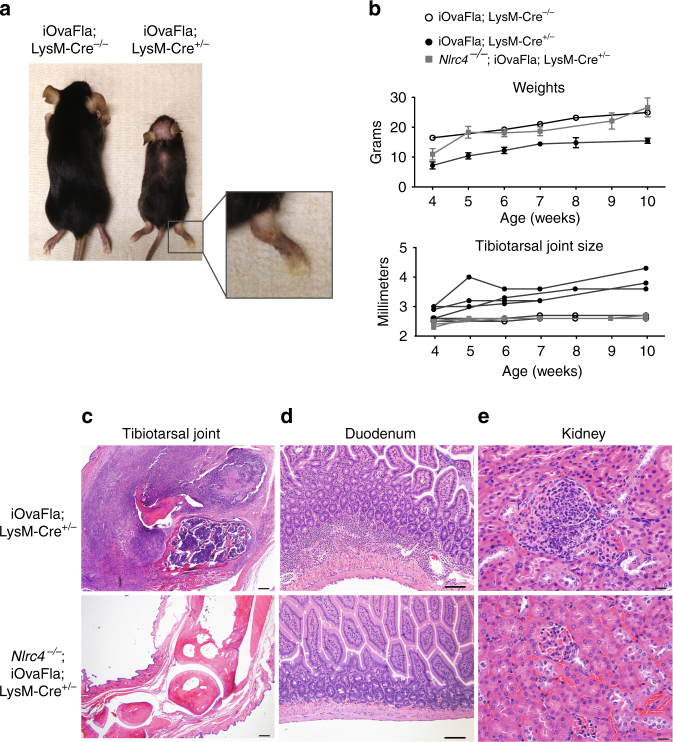
Myeloid-specific inflammasome activation generates systemic inflammation. Spontaneous inflammatory disease manifesting in the tibiotarsal joints **a**–**c**, the duodenum **d**, and kidney **e** of iOvaFla; LysM-Cre^+/−^ mice aged 10–14 weeks. **a** 14 week iOvaFla; LysM-Cre^+/−^ mice are runted and display joint disease compared to age and sex-matched iOvaFla; LysM-Cre^–^^/−^ mice. **b** Weight and tibiotarsal joint swelling as the mice age (*n* = 4–5 biological replicates per genotype). **c**–**e** Histology images showing **c** tibiotarsal joint, **d** duodenum, and **e** kidney damage in 10 week iOvaFla; LysM-Cre^+/−^ mice compared to *Nlrc4* ^−/−^; iOvaFla; LysM-Cre^+/−^ mice (representative images from three biological replicates; tibiotarsal joint at magnification ×40 and scale bar is 500 microns; duodenum at magnification ×600 and scale bar is 20 microns; kidney at magnification ×400 and scale bar is 20 microns). Data in **c**–**e** are representative of two independent experiments. Error bars are s.d.

We observed that iOvaFla; LysM-Cre^+/−^ mice were runted prior to weaning (Fig. 2a), and exhibited significantly lower body weights throughout life, compared to their age-matched Cre-negative littermates and the *Nlrc4* ^−/−^ control mice (Fig. 2b). Unexpectedly, the iOvaFla; LysM-Cre^+/−^ mice developed severe limb swelling, most noticeably in the tibiotarsal (heel) joint (Fig. 2a). Tibiotarsal joint swelling was 100% penetrant, although the age of onset varied, beginning as early as 4 weeks or as late as 10 weeks (Fig. 2b). Damage was more severe in the tibiotarsal joint as compared to the femorotibial (knee) joint (Supplementary Fig. 2a). In the tibiotarsal joint of iOvaFla; LysM-Cre^+/−^ mice, there was substantial neutrophilic infiltration with slight to severe bone and cartilage erosion in the joint. There were no histopathological findings in the joints of *Nlrc4* ^−/−^; iOvaFla; LysM-Cre^+/−^ (Fig. 2c) or in the LysM-Cre negative littermates (Supplementary Fig. 2b). Of note, some of the patients with an apparent auto-active NLRC4 mutation also experienced joint pain and one was diagnosed with psoriatic arthritis^19, 20, 23^.

In addition to joint damage, iOvaFla; LysM-Cre^+/−^ mice exhibited systemic inflammation. Notably, nearly all tissues examined exhibited significant neutrophilic infiltration. For example, pronounced neutrophilia was observed in sections of iOvaFla; LysM-Cre^+/−^ duodenums. This neutrophilic inflammation was not apparent in the absence of NLRC4 (Fig. 2d). Inflammation was distinguished by neutrophilic infiltration of the submucosa and lamina propria that also extended into the underlying muscularis externa. The inflammation was localised primarily to the duodenum and was not accompanied by damage to the surface epithelium (Fig. 2d). Interestingly, human patients with chronic NLRC4 auto-activation also have intestinal inflammation manifesting in the duodenum^20, 21^. There was also varying degrees of kidney damage in iOvaFla; LysM-Cre^+/−^ mice. Some mice had fibrin and neutrophil accumulation within glomeruli consistent with necrotizing glomerulonephritis, while others had some glomeruli with only thickened membranes. Once again, neither the *Nlrc4* ^−/−^; iOvaFla; LysM-Cre^+/−^ control (Fig. 2e) nor the LysM-Cre negative mice (Supplementary Fig. 2b) had pathological alterations. Consistent with hyperplasia, sick iOvaFla; LysM-Cre^+/−^ mice exhibited enlarged spleen and lymph nodes compared to their Cre negative littermates (Supplementary Fig. 2c). These data demonstrate that chronic myeloid-specific NAIP/NLRC4 inflammasome activation causes severe joint and systemic inflammation.

### Inflammasome-driven disease induces myeloid cell hyperplasia

**Fig. 3:**
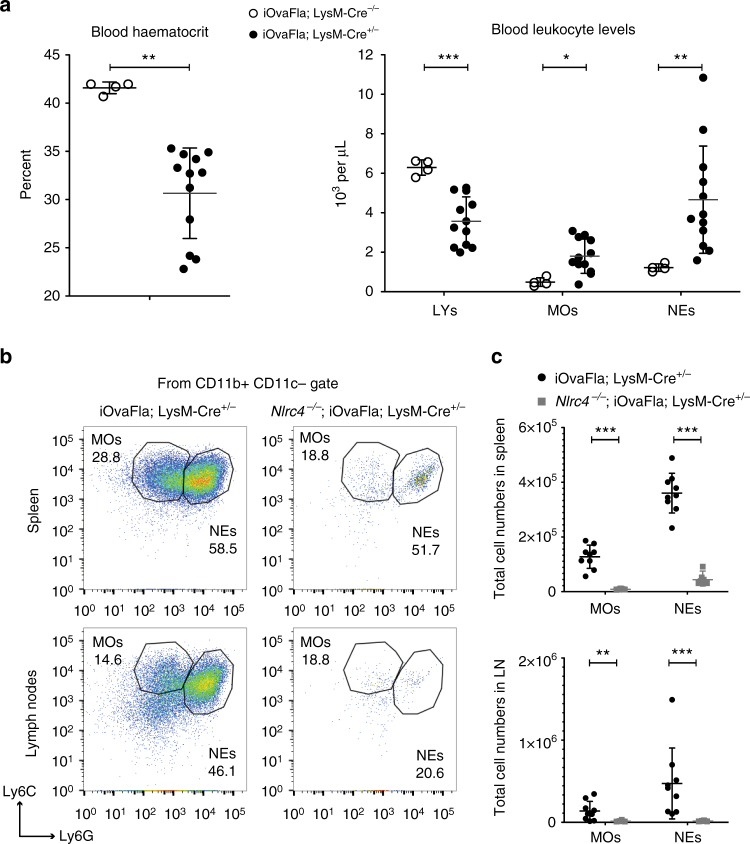
Inflammasome-driven disease leads to myeloid cell hyperplasia. **a** Haematocrit concentration and cell numbers from a complete blood count (CBC) performed on the blood of 10-week-old iOvaFla; LysM-Cre^+/−^ and iOvaFla; LysM-Cre^–/−^ mice. **b** Flow cytometry and **c** quantification of total monocytes (MO; CD11b^+^ Ly6C^Hi^ Ly6G^Lo^) and neutrophils (NE; CD11b^+^ Ly6C^Lo^ Ly6G^Hi^) from mice aged 10–16 weeks old. Flow cytometry plots are representative of three separate experiments. Dot plot percentages are from CD11b^+^ gate, and cell numbers are calculated from percentage of live cells (total cells). Data in **a** are representative of two independent experiments; Data in **b**, **c** are representative of three independent experiments. Error bars are s.d. Results were analysed with either a Mann–Whitney test or two-way ANOVA and Bonferroni post-tests; *p < 0.05, **p < 0.01, ***p < 0.001

To better understand inflammasome-driven pathology, we characterised haematopoietic cell populations in sick vs. control iOvaFla mice. Complete blood count (CBC) analysis of peripheral blood revealed that iOvaFla; LysM-Cre^+/−^ mice have decreased haematocrit levels compared to LysM-Cre^−/−^ littermates (Fig. 3a), consistent with anaemia of chronic disease (ACD) that has also been observed in other chronic inflammation models^37, 38^. Blood lymphocytes of iOvaFla; LysM-Cre^+/−^ mice were also decreased compared to healthy littermate controls. Finally, there were also significant (*p* < 0.05 and *p* < 0.01, respectively, by two-way ANOVA and Bonferroni post-test) increases in both monocytes and neutrophils in the peripheral blood of the sick LysM-Cre positive mice as compared to healthy littermates (Fig. 3a).

Flow cytometric analysis of spleen and pooled peripheral and mesenteric lymph nodes revealed that iOvaFla; LysM-Cre^+/−^ mice had increased levels of monocytes and neutrophils compared to the NLRC4-deficient control (Fig. 3b, c, Supplementary Fig. 3a). Although the OvaFla transgene should induce pyroptosis, the penetrance of OvaFla expression or OvaFla-induced pyroptosis is not complete (Supplementary Fig. 1), and it thus appears that cellular recruitment to tissues can overcome a comparatively low rate of pyroptosis. Lymphocyte populations were less dramatically affected, with B cell numbers decreasing modestly in the lymph nodes of iOvaFla; LysM-Cre^+/−^ as compared to *Nlrc4* ^−/−^; iOvaFla; LysM-Cre^+/−^ mice (Supplementary Fig. 3b, c). iOvaFla expression led to only slight changes in T cells levels in the spleen and lymph nodes, with perhaps a slight increase of T cells in the spleen as compared to control mice. No significant changes in T cell numbers were observed in the lymph nodes of iOvaFla; LysM-Cre^+/−^ mice compared to the control *Nlrc4* ^−/−^; iOvaFla; LysM-Cre^+/−^ mice (Supplementary Fig. 3d, e). Thus, iOvaFla expression in LysM-positive cells produces an increase in monocytes and neutrophils in an NLRC4-dependent manner, and only minor changes to the T and B cell numbers.

### Systemic cytokines increase in inflammasome-driven disease

**Fig. 4:**
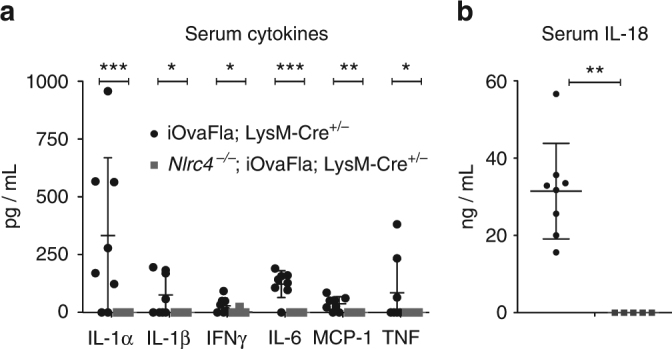
iOvaFla: LysM-Cre^+/−^ mice have increased levels of inflammatory cytokines. **a** BD Biosciences cytokine bead array and **b** IL-18 ELISA with serum from 16-week-old iOvaFla; LysM-Cre^+/−^ and *Nlrc4* ^−/−^; iOvaFla; LysM-Cre^+/−^ mice. Data are representative of three independent experiments. Error bars are s.d. Results were analysed with either a Mann–Whitney test or two-way ANOVA and Bonferroni post-tests; *p < 0.05, **p < 0.01, ***p < 0.001

Pro-IL-1β and pro-IL-18 are the two pro-cytokines known to be processed into their active and secreted forms by CASP1. Although pro-IL-18 is constitutively expressed in some cell types^39^, pro-IL-1β is often suggested to require a priming signal (‘signal 1’) for expression, prior to signals that activate CASP1 processing (‘signal 2’), as a safeguard to prevent inappropriate IL-1β production^26, 39^. However, exceptions to this simple model have been observed, including instances in which exogenous priming^40^ or CASP1 processing^41–43^ do not appear to be required for IL-1β production. Indeed, once inflammation is initiated, endogenous priming signals (e.g., inflammatory signals released from dying cells) may be sufficient to provide both signals 1 and 2 for IL-1β production. In our genetic system, no exogenous priming signal is provided, allowing us to assess whether exogenous provision of signal 2 alone is sufficient to initiate inflammatory cytokine production. We used a cytokine bead array to assay the amounts of IL-1α, IL-1β, IFNγ, IL-6, MCP-1 (also known as CCL2), and TNF in the serum of sick iOvaFla; LysM-Cre^+/−^ mice. All of the cytokines/chemokines were significantly (*p* < 0.05, *p* < 0.01 or *p* < 0.001, by two-way ANOVA and Bonferroni post-test) elevated compared to *Nlrc4* ^−/−^; iOvaFla; LysM-Cre^+/−^ mice (Fig. 4a). Interestingly, levels of IL-1β were also increased, confirming that constitutive expression or endogenous priming signals (e.g., from the microbiota) are sufficient to drive expression. IL-18 levels, as measured by ELISA, were also significantly (*p* < 0.01, by Mann–Whitney test) increased in iOvaFla; LysM-Cre^+/−^ mice (Fig. 4b). No cytokines or chemokines were detectable in *Nlrc4* ^−/−^; iOvaFla; LysM-Cre^+/−^ mice (Fig. 4a, b).

### ASC deficiency greatly delays auto-inflammatory disease

**Fig. 5:**
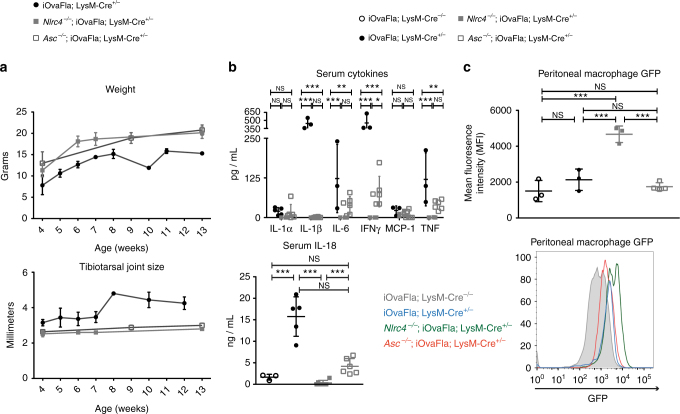
ASC deficiency ameliorates disease. **a** Weight and tibiotarsal joint swelling in iOvaFla; LysM-Cre^+/−^, *Nlrc4* ^−/−^; iOvaFla; LysM-Cre^+/−^, and *Asc* ^−/−^; iOvaFla; LysM-Cre^+/−^ mice of the indicated ages (*n* = 4–5 biological replicates per genotype). **b** Serum cytokines measured by a BD Biosciences CBA and IL-18 ELISA from 8-10-week-old mice. **c** Quantification and histogram of cellular GFP in peritoneal macrophages (CD11b^+^ F4/80^+^) of iOvaFla; LysM-Cre^−/−^, iOvaFla; LysM-Cre^+/−^, and *Nlrc4* ^−/−^; iOvaFla; LysM-Cre^+/−^, and *Asc* ^−/−^; iOvaFla; LysM-Cre^+/−^ mice. Data in **b**, **c** are representative of two independent experiments. Error bars are s.d. Results were analysed with a one-way or two-way ANOVA and Bonferroni post-tests; *p < 0.05, **p < 0.01, ***p < 0.001

The inflammasome adaptor protein ASC has a key function in mediating cytokine processing downstream of NLRC4 activation. Cytokine processing is largely dependent on ASC in vitro but less so in vivo^14, 44^. In order to determine whether ASC is required for disease in iOvaFla; LysM-Cre mice, we generated *Asc* ^−/−^; iOvaFla; LysM-Cre^+/−^ mice. Interestingly, disease was almost entirely ameliorated on the *Asc* ^−/−^ background, with normal weight gain and no joint inflammation evident through 10 weeks (Fig. 5a). *Asc* deficiency also severely reduced cytokine levels in the mice, though some cytokines, such as IL-6, IFNγ, and TNF were slightly increased on the *Asc* ^−/−^ background compared to the *Nlrc4* ^−/−^ control mice (Fig. 5b). Levels of GFP in *Asc* ^–*/–*^; iOvaFla; LysM-Cre^+/−^ resident peritoneal macrophages were comparable to those observed in *Asc* ^+^; iOvaFla; LysM-Cre^+/−^ macrophages, and significantly (*p* < 0.001, by two-way ANOVA and Bonferroni post-test) lower than those observed on *Nlrc4* ^–*/–*^; iOvaFla; LysM-Cre^+/−^ macrophages (Fig. 5c). These data imply that, as observed previously in vitro^14^, pyroptosis in vivo does not require ASC, and thus raise the possibility that cytokine release rather than pyroptosis is the major driver of disease.

### IL-1R signalling is necessary for the inflammatory phenotype

**Fig. 6:**
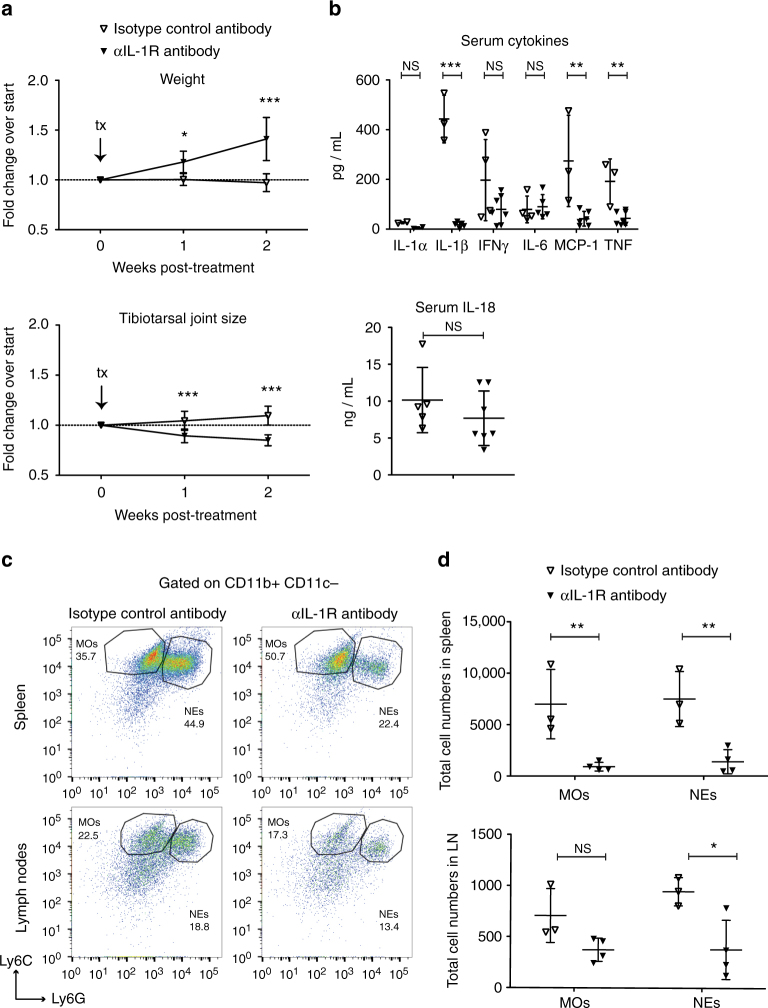
Blocking IL-1R relieves disease symptoms. iOvaFla; LysM-Cre^+/−^ mice with significant joint swelling at 4.0 mm were administered either anti-IL-1R blocking antibody or isotype control every 3–4 days for 2 weeks. **a **Their weights and tibiotarsal joints were measured during that time (*n* = 7 biological replicates per treatment). **b** Post-treatment serum cytokines measured by a BD Biosciences cytokine bead array and IL-18 ELISA. **c** Flow cytometry and **d** quantification of monocytes (MO; CD11b^+^ Ly6C^Hi^ Ly6G^Lo^) and neutrophils (NE; CD11b^+^ Ly6C^Lo^ Ly6G^Hi^). For each flow cytometry plot, the top row is from the spleen and the second row from peripheral and mesenteric lymph nodes. Each column represents a different treatment. Data in **b**–**d** are representative of three independent experiments. Error bars are s.d. Results were analysed with either a Mann–Whitney test or two-way ANOVA and Bonferroni post-tests; *p < 0.05, **p < 0.01, ***p < 0.001

After observing significant increases in IL-1 in sick iOvaFla mice (Fig. 4) and the decrease of IL-1 in the relatively healthy *Asc* ^–*/–*^; iOvaFla; LysM-Cre^+/−^ mice (Fig. 5b), we wanted to determine the importance of IL-1 signalling in the inflammatory phenotype. We therefore treated iOvaFla; LysM-Cre^+/−^ mice displaying tibiotarsal joint swelling with an αIL-1R blocking antibody every 3–4 days for 2 weeks. After treatment, tibiotarsal joint swelling decreased, and the mice gained weight compared to sick mice treated with isotype control antibody (Fig. 6a). The αIL-1R treated iOvaFla; LysM-Cre^+/−^ mice had significantly (*p* < 0.001, by two-way ANOVA and Bonferroni post-test) decreased amounts of IL-1β. The decreases in MCP-1 and TNF levels in treated mice were also statistically significant (*p* < 0.01, by two-way ANOVA and Bonferroni post-test) (Fig. 6b). Humans with auto-activating NLRC4 mutations still have elevated IL-18 after Anakinra treatment^21^. Consistent with these observations, we did not observe a statistically significant difference in IL-18 levels between control antibody and αIL-1R-treated mice (Fig. 6b). After αIL-1R antibody treatment, the sick iOvaFla; LysM-Cre^+/−^ mice also exhibited decreased levels of monocytes and neutrophils in both the spleen and lymph nodes when compared to control treated mice (Fig. 6c, d). Therefore, IL-1R signalling appears critical for development of the systemic inflammatory phenotype present in iOvaFla; LysM-Cre^+/−^ mice.

### NLRC4 activation in MRP8^+^ cells is sufficient for disease

**Fig. 7:**
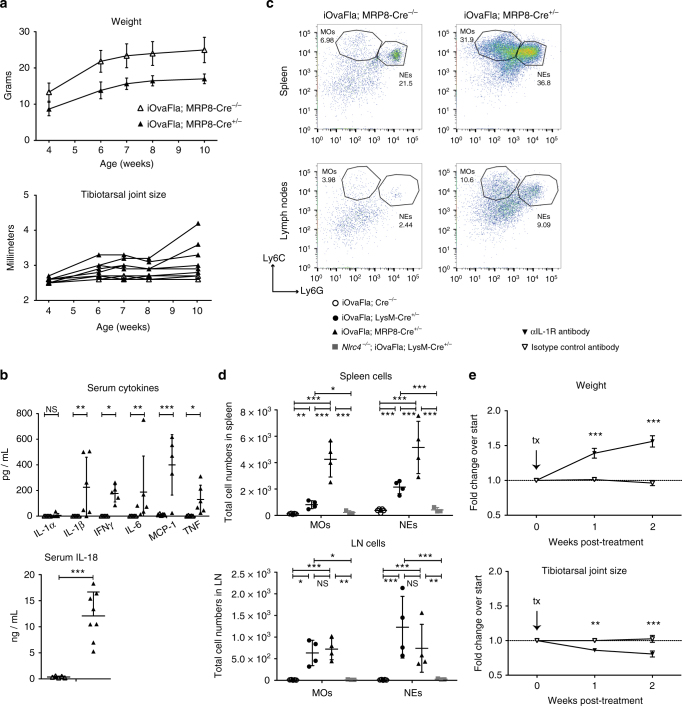
Neutrophil iOvaFla expression is sufficient for inflammatory disease. **a** Weight and tibiotarsal joint swelling as iOvaFla; MRP8-Cre^−/−^ and iOvaFla; MRP8-Cre^+/−^ mice age (*n* = 6 biological replicates per genotype). **b** Serum cytokines measured by a BD Biosciences cytokine bead array and IL-18 ELISA of 10–12-week-old mice. **c** Flow cytometry and **d** quantification of monocytes (MO; CD11b^+^ Ly6C^Hi^ Ly6G^Lo^) and neutrophils (NE; CD11b^+^ Ly6C^Lo^ Ly6G^Hi^). For each flow cytometry plot, the top row is from the spleen and the second row from peripheral and mesenteric lymph nodes. Each column represents a different genotype. **e** Weight and tibiotarsal joint measurements after OVA-Fla; MRP8-Cre^+/−^ mice were administered either anti-IL-1R blocking antibody or isotype control every 3–4 days for 2 weeks (*n* = 3 biological replicates per treatment). Data in **b**–**d** are representative of three independent experiments. Error bars are s.d. Results were analysed with either a Mann–Whitney test or two-way ANOVA and Bonferroni post-tests; *p < 0.05, **p < 0.01, ***p < 0.001

Neutrophilia is a prominent clinical feature of inflammasome-driven disease in our iOvaFla mice and in other mouse models^45, 46^ and humans^47–49^. Considering IL-1 is a powerful inducer of neutrophil recruitment to tissues, and given that we can alleviate disease by blocking IL-1R, it seems likely that neutrophils are responsible for a large portion of the inflammation. However, it is not clear whether inflammasome activation in neutrophils is sufficient to initiate disease, or whether inflammasome activation in other cell types releases IL-1, which then causes neutrophil recruitment. Therefore, we crossed the iOvaFla mice to MRP8-Cre transgenic mice, which expresses Cre primarily in neutrophils and in a small population of monocytes^29, 31, 32^. Surprisingly, the iOvaFla; MRP8-Cre^+/−^ mice almost fully recapitulated the severe disease phenotype of the iOvaFla; LysM-Cre^+^ mice. iOvaFla; MRP8-Cre^+/−^ mice are runted and develop joint swelling only slightly slower than iOvaFla; LysM-Cre^+/−^ mice (Figs. 7a, 2b). Moreover, iOvaFla; MRP8-Cre^+/−^ mice exhibit increased cytokine levels, with IL-18 being the most significantly (*p* < 0.001, by Mann–Whitney test) increased (Fig. 7b). Flow cytometry analysis demonstrated increases in monocytes and neutrophils in the spleen and lymph nodes, similar to what was observed in iOvaFla; LysM-Cre^+/−^ mice (Fig. 7c, d). In fact, iOvaFla; MRP8-Cre^+/−^ mice have even higher levels of monocytes and neutrophils in the spleen as compared to iOvaFla; LysM-Cre^+/−^ mice. There was no notable difference between the two genotypes in the numbers of inflammatory cells in the lymph nodes (Fig. 7 d). To determine whether IL-1 drives disease in iOvaFla; MRP8-Cre^+/−^ mice, we treated iOvaFla; MRP8-Cre^+/−^ with the same αIL-1R blocking antibody and protocol we used to treat iOvaFla; LysM-Cre^+/−^ mice (Fig. 6b). The treatment alleviated joint swelling and the mice gained weight compared to the isotype control treated mice, identical to LysM-Cre^+/−^ treated mice (Fig. 7e). These data suggest that inflammasome activation in neutrophils is an important contributor to IL-1-dependent inflammatory disease in vivo, though our data do not rule out additional contributions from monocytes as well.

To address whether iOvaFla expression in other myeloid cell types might also drive disease, we also crossed the iOvaFla mice to CD11c-Cre transgenic mice. CD11c-Cre mice express Cre in most dendritic cells, macrophages and monocytes, but unlike LysM-Cre mice, there is no expression in neutrophils^29, 33^. iOvaFla; CD11c-Cre^+/−^ mice exhibited some disease symptoms, including runting and elevation of monocytes and neutrophils in tissues. However, iOvaFla; CD11c-Cre^+/−^ mice exhibited less severe symptoms than iOvaFla; LysM-Cre or iOvaFla; MRP8-Cre mice. For example, iOvaFla; CD11c-Cre^+/–^ mice did not develop joint swelling (Supplementary Fig. 4a) even though monocyte and neutrophil numbers are mildly elevated (Supplementary Fig. 4b, c). Taken together, our results are consistent with the hypothesis that NLRC4 activation in MRP8^+^ cells is an especially potent driver of disease, though NLRC4 activation in CD11c^+^ cells can contribute to milder disease symptoms.

## Discussion

The question of whether activation of an endogenous wild-type inflammasome is a sufficient signal to trigger inflammatory disease remains unresolved. Several studies have used bacterial infections to stimulate inflammasome activation in vivo, but these infections are invariably accompanied by robust TLR activation, making it difficult to ascertain the specific and sufficient functions of inflammasome activation in vivo. Other studies have demonstrated clear pathological effects mediated by gain-of-function mutations in inflammasome genes^45, 46, 50^, but whether chronic activation of wild-type inflammasomes can also mediate disease is unresolved. For example, wild-type inflammasomes may be subject to feedback inhibitory mechanisms that are circumvented by the gain-of-function mutations observed in patients. In addition, patients may be exposed to other inflammatory stimuli, such as infections, that may contribute to inflammatory disease. Thus, to address whether chronic activation of endogenously expressed wild-type inflammasomes is sufficient to produce disease, we engineered a mouse that constitutively expresses cytosolic flagellin, a ligand for the NAIP/NLRC4 inflammasome, in the absence of any other contaminating exogenous stimuli. We found that selective NAIP/NLRC4 inflammasome activation in LysM^+^ positive cells is sufficient to cause a systemic inflammatory phenotype. iOvaFla; LysM-Cre^+/−^ mice are runted, exhibit elevated systemic cytokine levels, ACD, systemic neutrophilia, and obvious joint swelling. Although our genetically driven system for flagellin expression does not require or result in the provision of any additional exogenous priming signals, it is important to acknowledge that endogenous priming signals (e.g., from the microbiota) are inescapably present in our mice and may contribute to the disease symptoms we observe.

Disease in iOvaFla-expressing mice could be rescued by therapeutic blockade of the IL-1R. Disease could also be significantly reduced by crossing to the *Asc* ^−/−^ background. Interestingly, ASC is not believed to be essential for NAIP/NLRC4-induced pyroptosis^13, 14^, and indeed, ex vivo *Asc* ^−/−^; iOvaFla; LysM-Cre^+/−^ peritoneal macrophages still exhibited reduced expression of a co-expressed GFP, indicative that pyroptosis is still occurring in vivo. Similar to *Asc* deficiency, blockade of the IL-1R should also not prevent pyroptosis, yet this treatment significantly ameliorated disease. These lines of evidence suggest that chronic pyroptosis itself is not a sufficient driver of the inflammatory disease we observe after chronic inflammasome activation in vivo.

LysM-Cre is active in multiple cell types, including macrophages, monocytes, neutrophils and some dendritic cells^29^. Most studies of the inflammasome have been conducted in macrophages or monocytes, though a few studies have also indicated that neutrophils express functional inflammasomes^35, 36, 51, 52^. Interestingly, we found that expression of cytosolic flagellin in MRP8^+^ cells (mainly neutrophils) was sufficient to cause severe NLRC4-dependent systemic and joint inflammation, which was also rescued by blocking IL-1R. By contrast, chronic inflammasome activation mediated by CD11c-Cre (expressed in several cell types, including dendritic cells, tissue macrophages, and monocytes^29,33^ produced a milder disease with no joint pathology (Supplementary Fig. 4). Our results therefore uncover an unexpected function for inflammasome activation in MRP8^+^ cells in mediating systemic inflammatory disease.

Our results also indicate that IL-1 is a major driver of disease in our model. IL-1 is vital to the development of various auto-inflammatory human diseases, including inflammasome driven auto-inflammatory disorders. Patients with Deficiency of IL-1 receptor antagonist (DIRA) have systemic inflammation, including joint swelling and skin lesions. Lesion biopsies have exhibited excessive neutrophilia^49^. Patients with either rheumatoid arthritis (RA) or systemic juvenile idiopathic arthritis (sJIA) have increased serum IL-1 and joint neutrophil infiltration^53, 54^. In a mouse model of RA, neutrophil derived IL-1 is necessary for arthritis to develop^54^. Anaemia, leucocytosis, and arthritis are decreased in patients with sJIA treated with Anakinra^53^. Human inflammasomopathies have varying symptoms, some of which include flares of joint swelling and pain, uticaria and fever^19–23, 45^. When attempted, blocking IL-1R frequently improves the disorders. Consistent with our data, Canna et al.^21^ saw increased blood neutrophils during disease flares and decreased neutrophils after treating with IL-1 receptor antagonist. Interestingly, a patient carrying a V341A NLRC4 gain-of-function mutation was described that did not respond to anti-IL1 monotherapy^22^, but did respond to combination anti-IL-1 and anti-IL-18 therapy (anti-IL-18 monotherapy was not tested). Thus, IL-18 may also contribute to disease in specific scenarios.

Why might inflammasome activation in neutrophils be a particularly strong driver of auto-inflammatory disease? One possible explanation is that like other unusual cellular subpopulations^40^, these cells might circumvent the typical requirement for signal one (‘priming’) for expression of pro-IL-1β. Indeed, transcriptional profiling of specific neutrophil populations, including blood and liver neutrophils, indicates these cells express relatively high levels of pro-IL-1β at homoeostasis^55^. Another unusual feature of inflammasome activation in neutrophils is that it is reported in some cases to produce IL-1β release in the absence of pyroptosis^35^. Although pyroptosis is typically considered to be a pro-inflammatory form of cell death, our data suggest that pyroptosis is not necessarily pro-inflammatory or driving inflammatory disease in our model. On the contrary, it is possible that pyroptotic cell death is an important ‘self-limiting’ mechanism to prevent cells from sustained or prolonged release of IL-1β. Indeed, though we could see some evidence for NLRC4-dependent GFP loss from flagellin-expressing neutrophils, an indirect measure of pyroptosis, the pyroptotic loss of GFP in vivo was not nearly as prominent in neutrophils as compared to peritoneal macrophages, a cell population that has been shown to undergo robust pyroptosis^18^. Thus, we speculate that inflammasome activation (without co-incident pyroptosis) in neutrophils may drive pathology through sustained release of constitutively expressed IL-1β. Though there are few resident neutrophils in most tissues^56–58^, once inflammation is initiated, neutrophil recruitment to tissues may be enhanced and sustained by positive inflammatory feedback loops. Once these inflammatory feedback loops are established, it is possible they are maintained by IL-1 production by MRP8-Cre^+^, as well as Cre-negative cells.

Taken together, our results have identified inflammasome activation in MRP8^+^ cells, a population consisting primarily of neutrophils, as a sufficient driver of severe systemic inflammatory disease. In future studies, it will be of interest to determine with more specificity the role of neutrophils or monocytes as potent initiators of inflammation. In addition, it will be of interest to determine what role inflammasome activation in neutrophils has in the initiation and development of human inflammatory diseases.

## Methods

### Animals

All mice are bred and housed under specific pathogen-free conditions and fed a standard chow diet (Harlan irradiated laboratory animal diet). iOvaFla mice were generated by targeting the *Rosa26* locus for genomic insertion of a construct encoding a loxP‐flanked transcriptional STOP cassette upstream of the Ova-Fla fusion gene. An IRES-GFP was included downstream of the Ova-Fla insertion to mark cells in which the STOP cassette has been excised and Ova-Fla translated. Founders were crossed to ER-Cre^T2^ (Jax strain 008463), LysM-Cre (Jax strain 004781), MRP8-Cre (Jax strain 021614), CD11c-Cre (Jax strain 008068) and Villin-Cre (Jax strain 004586) transgenic lines. The strains were also crossed to *Nlrc4* ^*−/−*^ and *Asc* ^*−/−*^ to generate inflammasome-deficient control mice. *Nlrc4* ^*−/−*^ and *Asc* ^*−/−*^ animals were from V. Dixit (Genentech, South San Francisco, CA; Mariathasan et al.^13^).

Experiments were conducted on 10–16-week-old age- and generally sex-matched mice. Under isoflurane anaesthesia, mice were weighed weekly and callipers were used to measure tibiotarsal joint (heel) size. The mice were euthanized with CO_2_ with secondary cervical dislocation. All animal experiments and endpoints were approved by and performed in accordance with the regulations of the University of California Berkeley Institutional Animal Care and Use Committee.

### Bone marrow derived macrophages

Bone marrow was collected from femurs, and cells were differentiated into macrophages by culture in RPMI supplemented with cell supernatant from MCSF-transfected 3T3 cells (gift of B. Beutler) and 10% foetal bovine serum in a humidified incubator (37 °C, 5% CO_2_) for 7 days.

### Tissue histopathology

Tissues were collected from mice and fixed in 10% neutral buffered formalin. The fixed tissues were shipped to the University of Michigan Unit for Laboratory Animal Medicine for analysis. Soft tissues were processed in paraffin on an automated histology processor using standard IVAC protocols for mouse tissue. Hindlimb samples were decalcified in a commercial formic acid solution (ImmunoCal) for 3 days. After decalcification, the joints were rinsed and the femorotibial (knee) and tibiotarsal joints were isolated by cutting the bone proximal and distal to each joint. Joints were processed in paraffin as for soft tissues. Following processing, tibiotarsal (heel) joints were embedded in the lateral plane and the femorotibial (knee) joints and distal paw were embedded in the frontal plane, with compression using a manual histology tissue press. 4-micron thickness sections were cut on a rotary microtome and the sections were stained with haematoxylin and eosin on an automated tissue stainer.

Light microscopic evaluation was performed by a board-certified veterinary pathologist blinded to the groups at the time of sample evaluation. Representative photomicrographs were taken using an Olympus DP72 12.5-megapixel digital camera mounted to an Olympus BX45 light microscope and using the software provided by the manufacturer (cellSens Standard 1.7.1, Olympus Corporation). Photo processing and composite plate construction was performed in Adobe Photoshop CS2, version 9.0. Photo processing was confined to global adjustments of brightness, contrast, sharpness and image size that did not materially alter the interpretation of the image. Correction of peripheral lens distortion was performed if needed for low magnification photos.

### Complete blood counts

Blood was collected from mice either by saphenous bleeding or cardiac punctures followed by euthanasia. Blood was kept in EDTA-treated vacutainers (BD product 365974) and run on a Hemavet 850 at the University of California San Francisco Mouse Pathology Core.

### Flow cytometry

Spleens and lymph nodes were collected from euthanized mice, minced with scissors, and incubated in RPMI with 5% FBS containing collagenase VIII (1 mg/mL, Sigma) at 37 °C for 45 min. After straining the digested spleens and lymph nodes, single-cell suspensions were treated with ACK Lysing Buffer (Invitrogen A1049201) to lyse erythrocytes. Bone marrow flushed from femurs was also treated with ACK Lysing Buffer. Cells were washed and filtered through 40-micron nylon strainers (Fisher Scientific) and counted. Cells were blocked with anti-CD16/32 antibody (2.4G2) and stained for extracellular markers (Supplemental Table 1). The data were collected on Fortessa or ×20 flow cytometers (BD Biosciences), and analysis was performed using FlowJo 10 Software (Tree Star). Gating strategy is indicated in Supplementary Fig. 3.

### Immunoblot

To obtain peritoneal macrophages, 5 mL of PBS was injected into the peritoneal cavity then collected. Erythrocytes were lysed using ACK buffer (Invitrogen A1049201). 5 × 10^6^ cells were lysed in RIPA buffer supplemented with 1 mM PMSF and ×1 Roche Complete Protease Inhibitor Cocktail (Sigma 4693159001). Lysates were spun at max speed in an Eppendorf microfuge at 4 °C for 10 min and supernatants were mixed with SDS sample buffer (40% glycerol, 8% SDS, 2% 2-ME, 40 mM EDTA, 0.05% bromophenol blue, and 250 mM Tris-HCl (pH 6.8)), boiled for 5 min, and then separated by SDS-PAGE (Invitrogen 4–12% BisTris gel catalogue NP0335PK2). Separated proteins were transferred to Immobilon-FL PVDF membranes. Membranes were blocked with Odyssey blocking buffer (Licor 927-40000). Anti-GFP JL-8 antibody (Clontech 632380) and IRDye 800CW donkey anti-Mouse IgG (Licor 925-32212) were used. Densitometry was performed with Licor image studio lite.

### Serum cytokine measurements

To measure IL-18 by ELISA, Nunc Hi Affinity ELISA plates were coated with anti-IL-18 antibodies (MBL catalogue D047-3) at 1 μg/mL, blocked with PBS with 1% BSA (w/v). Serum was diluted 1:5 in PBS with 1% BSA (w/v). Secondary biotin conjugated goat antibodies to IL-18 (MBL catalogue D048-6) were used at 1:2000 in PBS with 1% BSA (w/v). Purified IL-18 standard was from Invivogen. Plates were developed with 1 mg/mL OPD (Sigma) in Citrate Buffer (PBS with 0.05 M NaH_2_PO_4_ and 0.02 M Citric acid) with a 3 M HCl acid stop after 10–15 min. Absorbance at 490 nm was measured on a SpectraMax M2. IL-1α, IL-1β, IFNγ, IL-6 and MCP-1 were measured with a custom BD Biosciences Cytokine Bead Array according to the BD Biosciences’ guidelines.

### Antibody treatments

Hamster IgG anti-mouse IL-1R antibody (mIL1R-M147) was obtained from Amgen. Ultra-LEAF Purified Armenian Hamster IgG Isotype Antibody from Biolegend (400940) was used for control injections. The mice were treated with 150 μg of each antibody via retro-orbital injections every 3–4 days for 2 weeks.

### Statistical analysis

Numerical data are expressed as the mean ± s.d. and were analysed with a Mann–Whitney test or a one-way or two-way ANOVA followed by a Bonferroni post-test. Figure legends indicate with analysis was used in the figure. 95% confidence was applied and data were considered significant at *p < 0.05, **p < 0.01, ***p < 0.001.

### Data availability

The authors declare that the main data supporting the findings of this study are available within the article and its Supplementary Information files. Extra data are available from the corresponding author upon reasonable request.

## Electronic supplementary material

Supplementary Information

## References

[1] Takeuchi O, Akira S (2010). Pattern recognition receptors and inflammation. Cell.

[2] Jones J, Vance RE, Dangl JL (2016). Intracellular innate immune surveillance devices in plants and animals. Science.

[3] Lamkanfi M, Dixit VM (2014). Mechanisms and functions of inflammasomes. Cell.

[4] Schroder K, Tschopp J (2010). The inflammasomes. Cell.

[5] Shi J (2015). Cleavage of GSDMD by inflammatory caspases determines pyroptotic cell death. Nature.

[6] Kayagaki N (2015). Caspase-11 cleaves gasdermin D for non-canonical inflammasome signalling. Nature.

[7] Jorgensen I, Miao EA (2015). Pyroptotic cell death defends against intracellular pathogens. Immunol. Rev..

[8] Bergsbaken T, Fink SL, Cookson BT (2009). Pyroptosis: host cell death and inflammation. Nat. Rev. Microbiol..

[9] Jorgensen I, Lopez JP, Laufer SA, Miao EA (2016). IL-1beta, IL-18, and eicosanoids promote neutrophil recruitment to pore-induced intracellular traps following pyroptosis. Eur. J. Immunol..

[10] Kofoed EM, Vance RE (2011). Innate immune recognition of bacterial ligands by NAIPs determines inflammasome specificity. Nature.

[11] Zhao Y (2011). The NLRC4 inflammasome receptors for bacterial flagellin and type III secretion apparatus. Nature.

[12] Lightfield KL (2008). Critical function for Naip5 in inflammasome activation by a conserved carboxy-terminal domain of flagellin. Nat. Immunol..

[13] Mariathasan S (2004). Differential activation of the inflammasome by caspase-1 adaptors ASC and Ipaf. Nature.

[14] Broz P, von Moltke J, Jones JW, Vance RE, Monack DM (2010). Differential requirement for Caspase-1 autoproteolysis in pathogen-induced cell death and cytokine processing. Cell Host. Microbe.

[15] von Moltke J, Ayres JS, Kofoed EM, Chavarria-Smith J, Vance RE (2013). Recognition of bacteria by inflammasomes. Annu. Rev. Immunol..

[16] Maltez VI, Miao EA (2016). Reassessing the evolutionary importance of inflammasomes. J. Immunol..

[17] Ayres JS, Trinidad NJ, Vance RE (2012). Lethal inflammasome activation by a multidrug-resistant pathobiont upon antibiotic disruption of the microbiota. Nat. Med..

[18] von Moltke J (2012). Rapid induction of inflammatory lipid mediators by the inflammasome in vivo. Nature.

[19] Kitamura A, Sasaki Y, Abe T, Kano H, Yasutomo K (2014). An inherited mutation in NLRC4 causes autoinflammation in human and mice. J. Exp. Med..

[20] Romberg N (2014). Mutation of NLRC4 causes a syndrome of enterocolitis and autoinflammation. Nat. Genet..

[21] Canna SW (2014). An activating NLRC4 inflammasome mutation causes autoinflammation with recurrent macrophage activation syndrome. Nat. Genet..

[22] Canna SW (2016). Life-threatening NLRC4-associated hyperinflammation successfully treated with IL-18 inhibition. J. Allergy Clin. Immunol..

[23] Kawasaki Y (2017). Identification of a high-frequency somatic NLRC4 mutation as a cause of autoinflammation by pluripotent cell-based phenotype dissection. Arth. Rheumatol..

[24] Hassane M (2017). Neutrophilic NLRP3 inflammasome-dependent IL-1beta secretion regulates the gammadeltaT17 cell response in respiratory bacterial infections. Mucosal Immunol..

[25] Fontana MF, Vance RE (2011). Two signal models in innate immunity. Immunol. Rev..

[26] Garlanda C, Dinarello CA, Mantovani A (2013). The interleukin-1 family: back to the future. Immunity.

[27] Rauch I (2016). NAIP proteins are required for cytosolic detection of specific bacterial ligands in vivo. J. Exp. Med..

[28] Rauch I (2017). NAIP-NLRC4 Inflammasomes coordinate intestinal epithelial cell expulsion with eicosanoid and IL-18 release via activation of caspase-1 and -8. Immunity.

[29] Abram CL, Roberge GL, Hu Y, Lowell CA (2014). Comparative analysis of the efficiency and specificity of myeloid-Cre deleting strains using ROSA-EYFP reporter mice. J. Immunol. Methods.

[30] Clausen BE, Burkhardt C, Reith W, Renkawitz R, Förster I (1999). Conditional gene targeting in macrophages and granulocytes using LysMcre mice. Transgenic Res..

[31] Lagasse E, Weissman IL (1994). bcl-2 inhibits apoptosis of neutrophils but not their engulfment by macrophages. J. Exp. Med..

[32] Passegue E, Wagner EF, Weissman IL (2004). JunB deficiency leads to a myeloproliferative disorder arising from hematopoietic stem cells. Cell.

[33] Caton ML, Smith-Raska MR, Reizis B (2007). Notch-RBP-J signaling controls the homeostasis of CD8- dendritic cells in the spleen. J. Exp. Med..

[34] Soriano P (1999). Generalized lacZ expression with the ROSA26 Cre reporter strain. Nat. Genet..

[35] Chen KW (2014). The neutrophil NLRC4 inflammasome selectively promotes IL-1beta maturation without pyroptosis during acute Salmonella challenge. Cell Rep..

[36] Ryu JC (2016). Neutrophil pyroptosis mediates pathology of P. aeruginosa lung infection in the absence of the NADPH oxidase NOX2. Mucosal Immunol..

[37] Mouchess ML (2011). Transmembrane mutations in Toll-like receptor 9 bypass the requirement for ectodomain proteolysis and induce fatal inflammation. Immunity.

[38] Weiss G, Goodnough LT (2005). Anemia of chronic disease. N. Engl. J. Med..

[39] Puren AJ, Fantuzzi G, Dinarello CA (1999). Gene expression, synthesis, and secretion of interleukin 18 and interleukin 1beta are differentially regulated in human blood mononuclear cells and mouse spleen cells. Proc. Natl Acad. Sci. USA.

[40] Franchi L (2012). NLRC4-driven production of IL-1beta discriminates between pathogenic and commensal bacteria and promotes host intestinal defense. Nat. Immunol..

[41] Karmakar M, Sun Y, Hise AG, Rietsch A, Pearlman E (2012). Cutting edge: IL-1beta processing during Pseudomonas aeruginosa infection is mediated by neutrophil serine proteases and is independent of NLRC4 and caspase-1. J. Immunol..

[42] Mayer-Barber KD (2010). Caspase-1 independent IL-1beta production is critical for host resistance to mycobacterium tuberculosis and does not require TLR singaling in vivo. J. Immunol..

[43] Lukens JR (2014). Critical role for inflammasome-independent IL-1beta production in osteomyelitis. Proc. Natl Acad. Sci. USA.

[44] Patankar YR, Mabaera R, Berwin B (2015). Differential ASC requirements reveal a key role for neutrophils and a noncanonical IL-1β response to Pseudomonas aeruginosa. Am. J. Physiol. Cell Mol. Physiol..

[45] Brydges SD (2009). Inflammasome-mediated disease animal models reveal roles for innate but not adaptive immunity. Immunity.

[46] Masters SL (2012). NLRP1 inflammasome activation induces pyroptosis of hematopoietic progenitor cells. Immunity.

[47] Hoffman HM (2004). Prevention of cold-associated acute inflammation in familial cold autoinflammatory syndrome by interleukin-1 receptor antagonist. Lancet.

[48] Haas N, Kuster W, Zuberbier T, Henz BM (2004). Muckle-Wells syndrome: clinical and histological skin findings compatible with cold air urticaria in a large kindred. Br. J. Dermatol..

[49] Aksentijevich I (2009). An autoinflammatory disease with deficiency of the interleukin-1-receptor antagonist. N. Engl. J. Med..

[50] Brydges SD (2013). Divergence of IL-1, IL-18, and cell death in NLRP3 inflammasomopathies. J. Clin. Invest..

[51] Cho JS (2012). Neutrophil-derived IL-1beta is sufficient for abscess formation in immunity against Staphylococcus aureus in mice. PLoS Pathog..

[52] Karmakar M (2015). Neutrophil IL-1beta processing induced by pneumolysin is mediated by the NLRP3/ASC inflammasome and caspase-1 activation and is dependent on K + efflux. J. Immunol..

[53] Pascual V, Allantaz F, Arce E, Punaro M, Banchereau J (2005). Role of interleukin-1 (IL-1) in the pathogenesis of systemic onset juvenile idiopathic arthritis and clinical response to IL-1 blockade. J. Exp. Med..

[54] Chou RC (2010). Lipid-cytokine-chemokine cascade drives neutrophil recruitment in a murine model of inflammatory arthritis. Immunity.

[55] Heng TS, Painter MW (2008). The Immunological Genome Project: networks of gene expression in immune cells. Nat. Immunol..

[56] Peters AM (1998). Just how big is the pulmonary granulocyte pool?. Clin. Sci..

[57] Kreisel D (2010). In vivo two-photon imaging reveals monocyte-dependent neutrophil extravasation during pulmonary inflammation. Proc. Natl Acad. Sci. USA.

[58] Kolaczkowska E, Kubes P (2013). Neutrophil recruitment and function in health and inflammation. Nat. Rev. Immunol..

