# Supplementary material for: NAIP/NLRC4 inflammasome activation in MRP8+ cells is sufficient to cause systemic inflammatory disease
Source: Nat Commun. 2017 Dec 20;8:2209. doi: 10.1038/s41467-017-02266-w (PMC5738432; doi:10.1038/s41467-017-02266-w)
Supplement: Supplementary file 1 — Supplementary Information [file 41467_2017_2266_MOESM1_ESM.pdf]

Supplementary Fig. 1

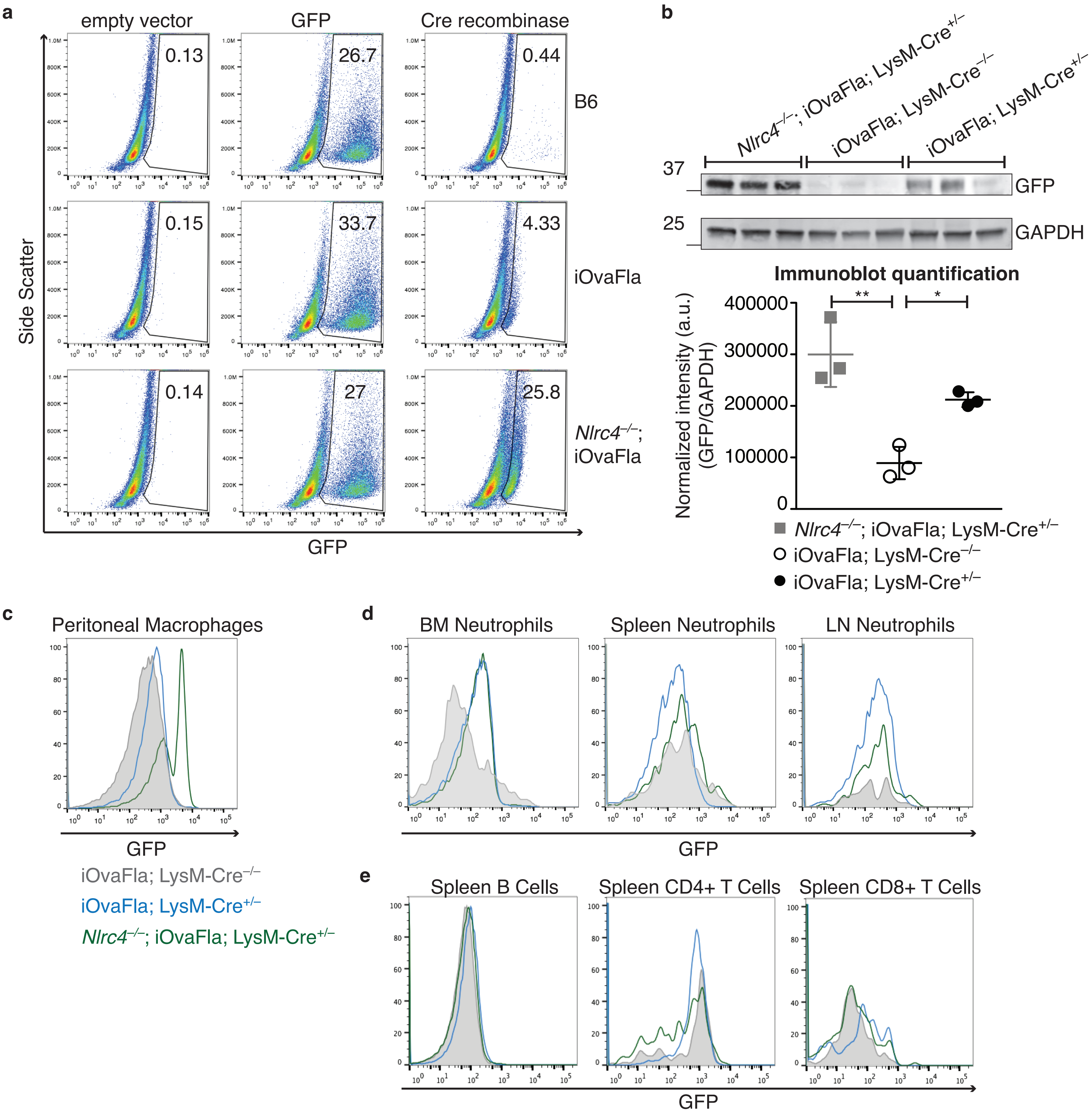

Supplementary Fig. 1. Genetic system for cell type-specific inflammasome activation in vivo. (a) Retroviral transduction of B6, iOvaFla$^{+/+}$, and *Nlrc4*$^{-/-}$; iOvaFla$^{+/+}$ BMMs with either an empty MSCV vector, MSCV vector containing only GFP, or MSCV vector expressing Cre Recombinase. **(b)** GFP immunoblot and quantification performed on *ex vivo* peritoneal macrophages. Quantification was performed using Licor image studio light. GFP histograms of **(c)** peritoneal macrophages, **(d)** neutrophils from the bone marrow, spleen, or lymph nodes, and **(e)** splenic B cells (CD19^{+} TCRb^{-}), CD4^{+} (TCRb^{+} C19^{-} CD4^{+}), and CD8^{+} T cells (TCRb^{+} C19^{-} CD8^{+}). Data in (c - e) are representative from n=3 biological replicates and (a - d) two or (e) three independent experiments. Error bars are s.d. Results were analyzed with an one-way ANOVA and Bonferroni post-tests; * = $p < 0.05$, ** = $p < 0.01$.

Supplementary Fig. 2

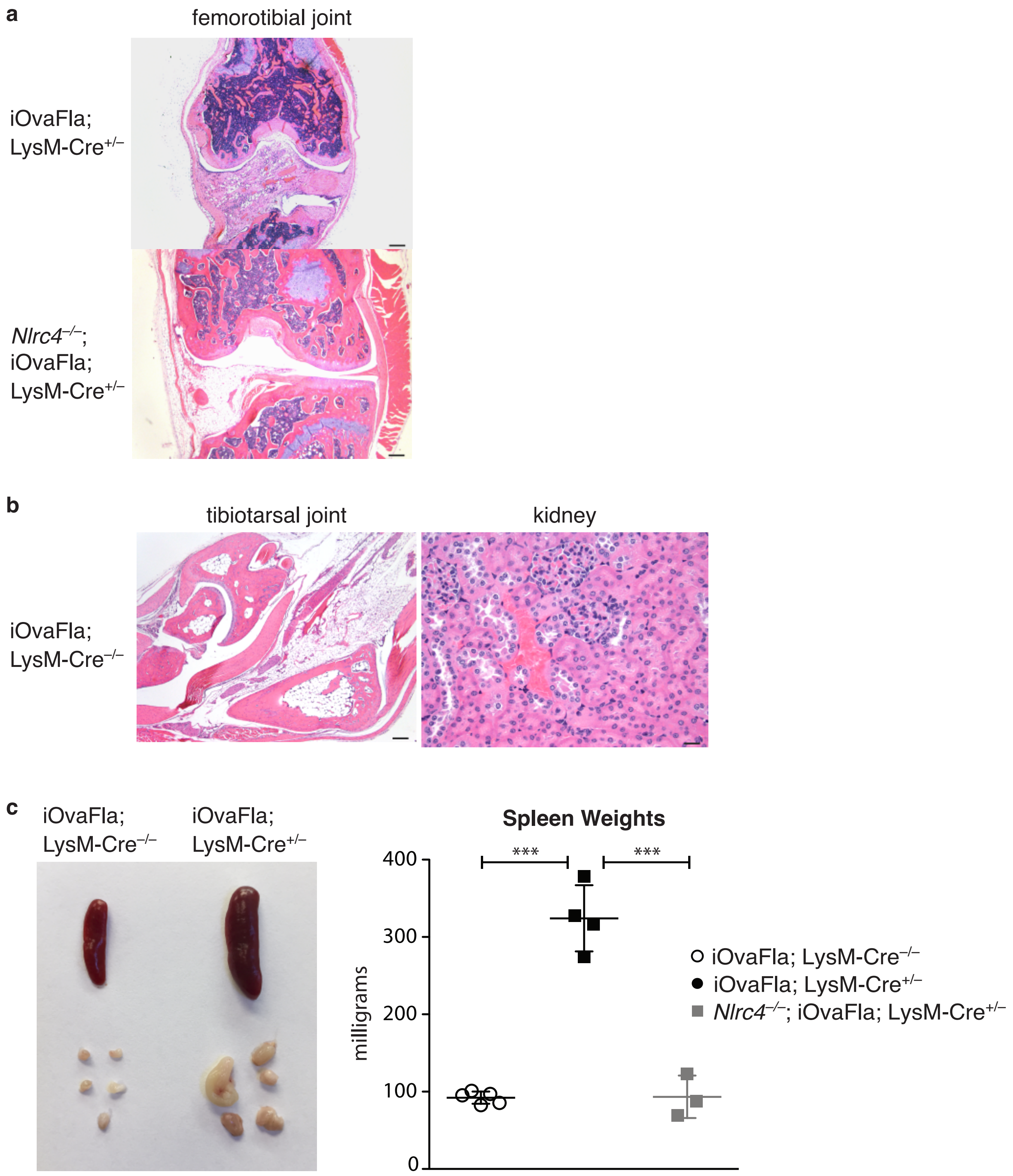

Supplementary Fig. 2. Systemic inflammation in iOvaFla$^{+/-}$; LysM-Cre$^{+/-}$ mice. Histology images showing (a) femorotibial joint in 10 week iOvaFla; LysM-Cre$^{+/-}$ mice compared to *Nlrc4*$^{-/-}$; iOvaFla; LysM-Cre$^{+/-}$ mice (representative from 3 biological replicates) and **(b)** tibiotarsal joint and kidney of iOvaFla; LysM-Cre$^{-/-}$ mice (representative from 2 biological replicates; femorotibial and tibiotarsal joints at magnification X40 and scale bar is 500 microns; kidney at magnification X400 and scale bar is 20 microns). **(c)** Spleen and lymph nodes of iOvaFla; LysM-Cre$^{-/-}$ and iOvaFla; LysM-Cre$^{+/-}$ mice and spleen weights from iOvaFla; LysM-Cre$^{-/-}$, iOvaFla; LysM-Cre$^{+/-}$, *Nlrc4*$^{-/-}$; iOvaFla; LysM-Cre$^{+/-}$ mice (representative from two separate experiments). Error bars are s.d. Results were analyzed with a one-way ANOVA and Bonferroni post-tests; *** = $p < 0.001$.

Supplementary Fig. 3

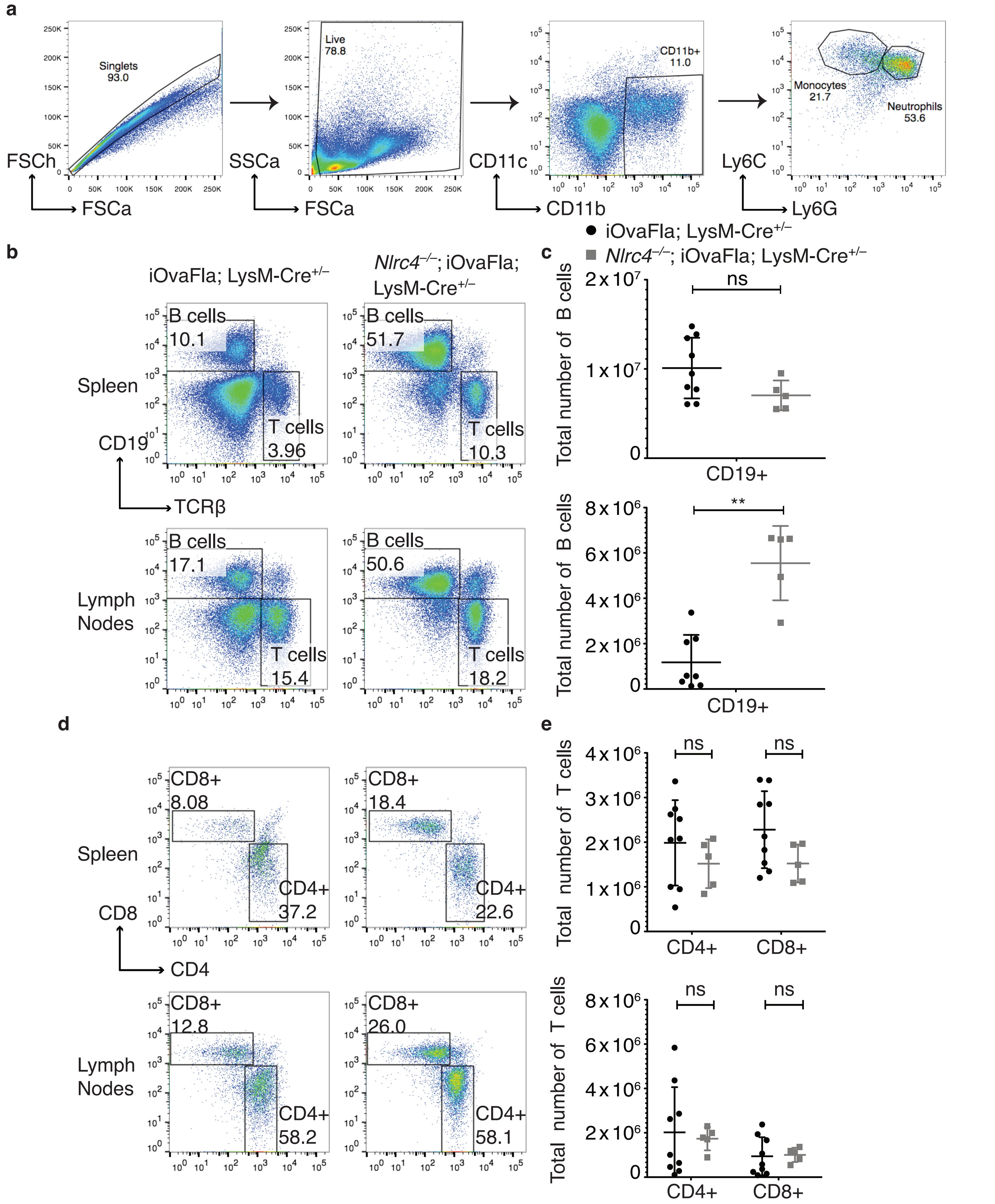

Supplementary Fig. 3. Lymphocyte populations mostly unchanged in iOvaFla; LysM-Cre$^{+/-}$ mice. (a) Gating strategy to determine monocytes and neutrophils. Example is an iOvaFla; LysM-Cre$^{+/-}$ mouse. **(b)** Flow cytometry and **(c)** quantification of B cells (CD19^{+} TCRb^{-}). **(d)** Flow cytometry and **(e)** quantification of CD4^{+} (TCRb^{+} C19^{-} CD4^{+}) and CD8^{+} T cells (TCRb^{+} C19^{-} CD8^{+}). b – e are representative of 3 independent experiments. Error bars are s.d. Results were analyzed with either a Mann-Whitney t-test or two-way ANOVA and Bonferroni post-tests; * = $p < 0.05$, ** = $p < 0.01$, *** = $p < 0.001$.

Supplementary Fig. 4

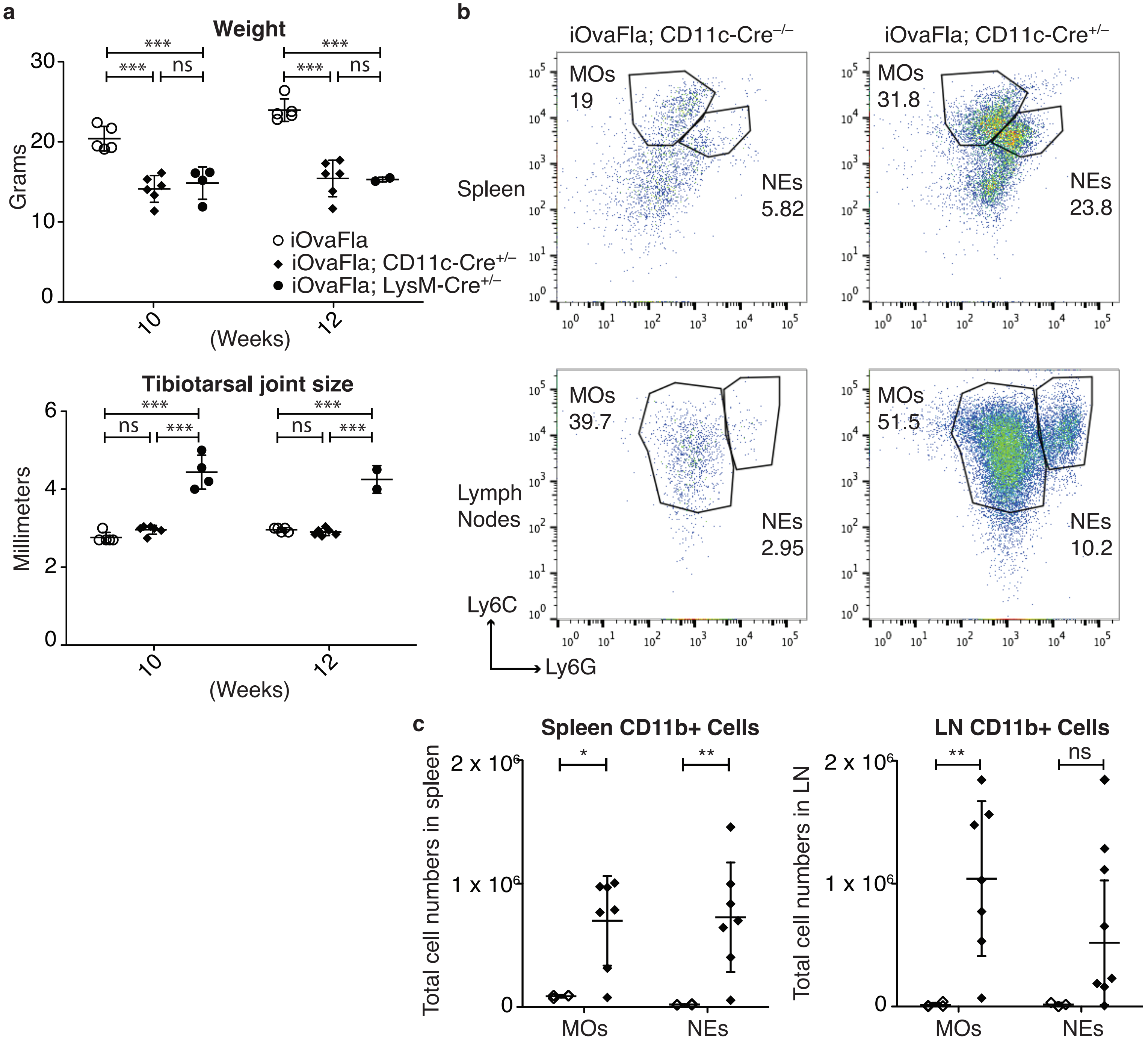

Supplementary Fig. 4. iOvaFla; CD11c-Cre$^{+/-}$ mice have a less severe inflammatory phenotype. (a) Weight and tibiotarsal joint swelling as iOvaFla; Cre$^{-/-}$, iOvaFla; CD11c-Cre$^{+/-}$, and iOvaFla; LysM-Cre$^{+/-}$ mice age. **(b** and **c)**. Flow cytometry plots and cell number graphs are a compilation of two separate experiments of mice aged 12-20 weeks old. For the flow cytometry plot, the top row is from the spleen and the second row from peripheral and mesenteric lymph nodes. Each column represents a different genotype. **(b)** Flow cytometry and **(c)** quantification of monocytes (MO; CD11b^{+} Ly6C^{Hi} Ly6G^{Lo}) and neutrophils (NE; CD11b^{+} Ly6C^{Lo} Ly6G^{Hi}). Data in (b, c) are representative of two independent experiments. Error bars are s.d. Results were analyzed with a two-way ANOVA and Bonferroni post-tests; * = $p < 0.05$, ** = $p < 0.01$, *** = $p < 0.001$.

Supplemental Table 1

Antibody	Clone	Used at	Supplier
Anti-CD11b PB	M1/70	1:250	eBiosciences 48-0112
Anti-CD11c APC	N418	1:250	eBiosciences 17-0114
Anti-Ly6G PE Cy7	1A8	1:250	Biolegend 127618
Anti-Ly6C PerCP Cy5.5	HK1.4	1:250	eBiosciences 45-5932
Anti-MHCII PE	M5/114.15.2	1:500	eBiosciences 12-5321
Anti-CD19 PE	eBio1D3	1:250	eBiosciences 12-0193
Anti-CD3 APC	145-2C11	1:250	eBiosciences 17-0031
Anti-CD4 PerCP e710	GK1.5	1:250	eBiosciences 25-0041
Anti-CD8 APC Cy7	53-6.7	1:250	eBiosciences 47-0081

Table listing all the flow cytometry antibodies used, the antibody clone, concentration used, and the supplier and catalogue number.
